# Effects of long-term tillage on soil nitrogen transformation, nitrogen fractions, and wheat yield

**DOI:** 10.3389/fpls.2026.1847545

**Published:** 2026-05-28

**Authors:** Qingyuan Wang, Yu Shi, Zhenwen Yu, Yongli Zhang, Zhen Zhang

**Affiliations:** 1 National Key Laboratory of Wheat Improvement, Ministry of Agriculture, Agricultural College, Shandong Agricultural University, Taian, Shandong, China; 2 Key Laboratory of Crop Ecophysiology and Farming System, Ministry of Agriculture, Agricultural College, Shandong Agricultural University, Taian, Shandong, China

**Keywords:** 15N tracer, crop yield, soil nitrogen fractions, soil nitrogen transformation, tillage practices

## Abstract

The response of soil nitrogen (N) transformation processes and N supply capacity to tillage practices is crucial for reducing soil N loss and promoting sustainable agricultural development. However, the comprehensive research and driving mechanism of how long-term positioning tillage methods affect wheat yield through soil nitrogen transformation, soil nitrogen supply capacity, and soil nitrogen loss are still unclear. A long-term field experiment was initiated in 2007 (lasting 16 years) with four tillage treatments: perennial plowing tillage (PT), perennial rotary tillage (RT), perennial strip rotary tillage (ST), and subsoiling every two years combined with strip rotary tillage (STS). In 2022, the changes of soil N transformation intensity, soil N-transforming enzyme activity, NH_3_ volatilization, N_2_O emission and soil N fractions in wheat fields were measured at different wheat growth stages. Additionally, ^15^N isotope tracer technology was used to explore the N absorption and utilization of plants under different tillage practices. Our results demonstrated that, compared to PT, RT, and ST, the STS treatment significantly increased the intensity of soil nitrogen fixation, ammonification and nitrification at different growth stages of wheat. It also increased soil urease and protease activities while reducing denitrification intensity, nitrate reductase, and nitrite reductase activities, consequently decreasing cumulative N_2_O emissions. Furthermore, the STS treatment significantly increased soil total nitrogen, particulate organic nitrogen, soluble organic nitrogen, microbial biomass nitrogen, ammonium nitrogen, and nitrate nitrogen contents. The ^15^N tracer technology showed that STS treatment could promote the absorption of N fertilizer and reduce the loss of N fertilizer. In addition, STS significantly increased the grain yield by 8.62%-20.44% compared with other treatments, with better yield stability. Through partial least squares method, it was found that STS significantly promotes N conversion and N pool composition, indirectly promotes N absorption, thereby promoting wheat yield increase, while suppressing cumulative N_2_O emissions and reducing N loss. Overall, the STS treatment enhanced soil N transformation, reduced soil N loss, provided sufficient N support for plant growth, promoted N uptake, and achieved the highest grain yield. Therefore, STS represents a sustainable tillage method for reducing soil N loss and improving crop yield.

## Graphical Abstract

**Figure:**
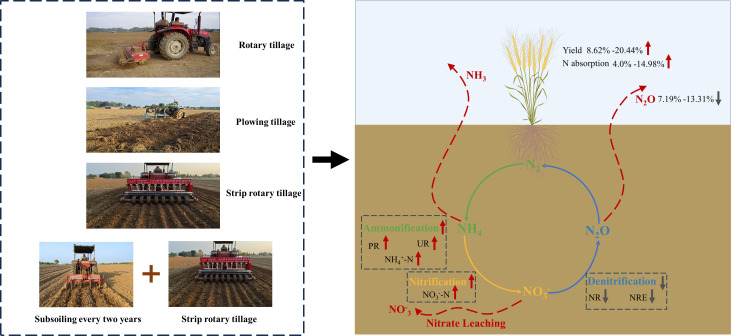
The abstract should ideally be structured according to the IMRaD format (Introduction, Methods, Results and Discussion). Provide a structured abstract if possible. If your article has been copyedited by us, please provide the updated abstract based on this version.

## 1 Introduction

The Huang-Huai-Hai region is an important production area of winter wheat in China, and its output accounts for more than 70% of the total output of the country, which plays an extremely important role in ensuring national food security (Wu et al., 2023; Zhao et al., 2024). However, the long-term adoption of rotary tillage in this region has led to the formation of a compacted plow pan, which restricts root penetration and consequently limits crop yield potential (Li et al., 2024). Furthermore, continuous years of ploughing have also produced problems such as soil erosion, water loss and reduced economic benefits. Additionally, frequent soil tillage operations in this region have accelerated the decomposition of soil organic matter, leading to significant N losses and posing challenges to sustainable agricultural development (Yuan et al., 2024). Therefore, the selection of appropriate tillage practices is essential to promote the sustainable development of agriculture in the region.

Conservation tillage is a crucial management practice that enhances soil productivity and sustainability by minimizing soil erosion and runoff while improving soil moisture and nutrient retention through reduced soil disturbance and straw mulching (Li et al., 2021; Shen et al., 2022). The transition from conventional tillage to conservation tillage may significantly influence nutrient cycling in complex ways, particularly concerning N transformation, supply, and loss, through alterations in substrate availability and soil microenvironments (Laine et al., 2018). Consequently, characterising post-tillage N transformation processes and understanding supply mechanisms are crucial for optimising N management practices within agroecosystems. In no tillage systems, organic residues accumulate more in the soil surface, slowing down the decomposition of organic matter, which results in a lower nitrification rate in no tillage treatment compared to tillage (Li et al., 2015). With the reduction of soil disturbance, no tillage and less tillage under straw mulching reduced soil organic N mineralization and increased N fixation in the soil, which may lead to reduced accumulation of soil mineral N and lower N leaching potential (Nevins et al., 2021; Grados et al., 2022; Yuan et al., 2022). Although net mineralization and nitrification rates have been used to evaluate the impact of tillage practices on soil N transformation (Liu et al., 2017; Jahangir et al., 2020), the specific mechanisms of long-term tillage practices on soil N transformation are still poorly understood. Meanwhile, few studies have considered the integrated effects of soil N transformation processes as a whole under long-term positional tillage practices. This crucial knowledge gap limits our ability to accurately measure N dynamics and formulate effective strategies to reduce N loss. In this process, N_2_O emission and NH_3_ volatilization as the products of the N transformation process, resulting in N loss that cannot be ignored (Deng et al., 2023). For example, Subsoiling can reduce the loss of soil total active N, but increase the risk of ammonia volatilization (Wang et al., 2020; Farooq et al., 2022).

Soil N-transforming enzyme activity is involved in the process of soil N transformation, which can characterize the supply and transformation ability of N in soil and reflect the absorption and utilization of N by crops to a certain extent (Li and Wang, 2023; Wang et al., 2021).

Soil urease and protease are the key enzymes in soil ammonification process, which hydrolyze urea and protein into ammonium nitrogen and amino acid, respectively, and affect the conversion of ammonia to NH_3_ (Huang et al., 2019; Greenfield et al., 2020). Nitrate reductase and nitrite reductase can be directly involved in the denitrification process of soil. The tillage method has a significant impact on soil N-transforming enzymes. For example, deep tillage combined with straw returning can create a favorable soil environment that enhances soil N transforming enzyme activity and nutrient availability by increasing soil moisture and microbial activity (Fahad et al., 2024). A meta-analysis found that the effect of soil N-transforming enzyme activity on tillage practices is influenced by soil depth, crop type, and tillage years (Wen et al., 2023). However, the mechanism by which soil N-transforming enzymes affect the process of soil N transformation under long-term cultivation conditions is still unclear.

The process of soil N transformation is closely related to the form of soil organic N components. Particulate organic nitrogen (PON), microbial biomass nitrogen (MBN) and soluble organic nitrogen (SON), as important components of unstable soil organic N, can participate in the process of soil N transformation in a short time, and are the intermediate N pools for stable organic N to mineral N transformation (Wander, 2004; Liu et al., 2021; Sainju and Lenssen, 2011). This helps to accurately describe the accumulation and sensitivity of soil N components to tillage responses, thus enabling a deeper understanding of soil N transformation dynamics and is of great significance for reducing N loss. Research shows that subsoiling treatment under straw returning can improve soil N supply capacity, which has great potential in increasing mineral N supply and maintaining stable organic N (Yu et al., 2024). The depth and intensity of soil disturbance caused by different tillage methods are different, resulting in the change of total N content in different soil layers after tillage, which has an important impact on fixing soil N and reducing the loss caused by soil N leaching and volatilization (Xue et al., 2015; Lv et al., 2023). However, information on how long-term farming affects the N composition of each soil layer at different growth stages is very limited. The response of organic nitrogen components to soil N transformation under long-term cultivation strategies has not been evaluated. In addition, ^15^N tracer technology provides valuable information on soil N dynamics and fate, which is important for accurately grasping soil N supply and reducing N losses (Scheer and Rütting, 2023; Dong et al., 2022).

At present, optimized tillage and crop cover management strategies have been developed in the Huang-Huai-Hai region to maintain crop yields and improve fertilizer utilization efficiency. However, the comprehensive research and driving mechanism of how long-term positioning tillage affect wheat yield through soil N transformation, soil nitrogen supply capacity, and soil nitrogen loss are still unclear. This study combines indoor cultivation, field experiments, and ^15^N tracing technology to investigate the effects of long-term fixed tillage methods on soil N transformation, soil N components, and soil N loss. We assume that compared with other tillage methods, the combination of subsoiling every two years combined with strip rotary tillage can promote soil N transformation, thereby improving N supply and absorption capacity, further reducing soil N loss, promoting plant N absorption, and achieving the highest grain yield. The objectives of this study were (1) to study the long-term effects of tillage practices on soil N transformation processes (2) to clarify the effects of long-term positioning tillage practices on soil N fractions in soil layers at different fertility periods of wheat (3) to identify the driving pathways for N absorption and yield improvement, select the optimal tillage system to reduce N loss and improve wheat yield. The research results will provide a sustainable farming method for reducing soil N loss and increasing yield in the Huang-Huai-Hai region, and provide theoretical basis and practical guidance for sustainable management in the area.

## 2 Materials and methods

### 2.1 Experimental site

**Figure 1:**
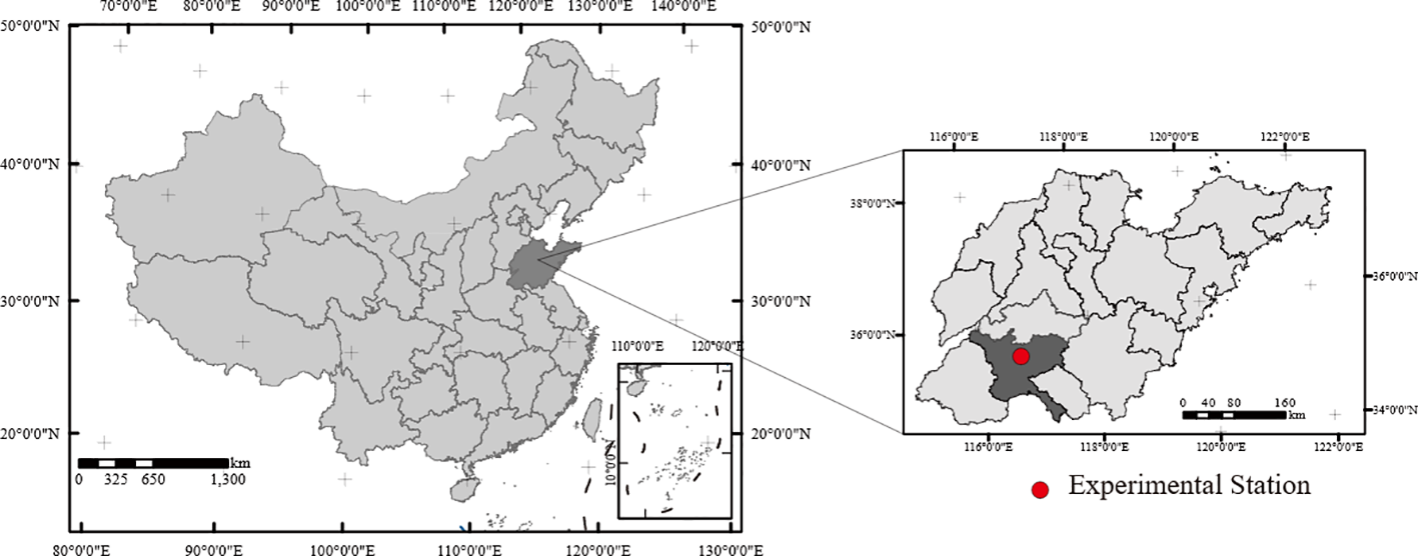
Schematic diagram of experimental testing location.

**Figure 2:**
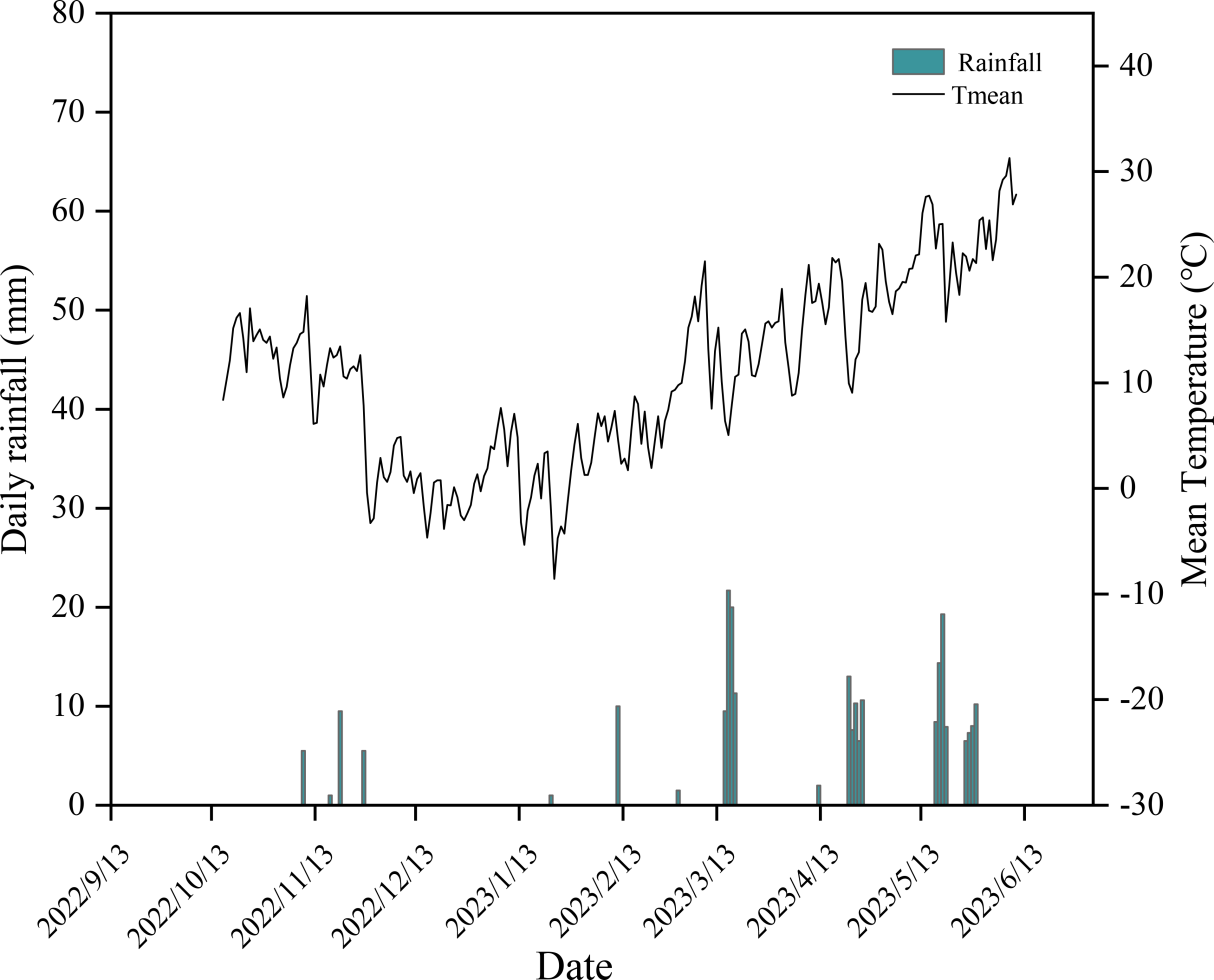
Daily rainfall (bars) and average temperature (line) during the wheat growing seasons of 2022–2023.

The field trial was established in 2007 in Xiaomeng Town (35°40′N, 116°41′E), Yanzhou District, Jining City, Shandong Province. The specific test location is shown in **Figure 1**. The soil type of the experimental field was Haplic luvisols, which has a pH value of 7.6 and consists of 29.6% clay, 37.3% silt and 33.1% sand. The previous crop was maize. The site has a temperate continental monsoon climate with a mean temperature of 13.6 °C and a mean annual precipitation of 733 mm. The specific meteorological data is shown in **Figure 2**. The basal ground conditions before positioning in 2007 and before sowing in 2022 are shown in **Table 1**.

**Table 1:** Soil nutrient content in 0–20 cm soil layer.

Year	Treatment	Organic matter (g kg^-1^)	Total N (g kg^-1^)	Available N (mg kg^-1^)	Available P (mg kg^-1^)	Available K (mg kg^-1^)
2007~2008	PT/RT/ST/STS	14.1	1.00	103.13	23.30	121.58
2022~2023	PT	14.23	1.08	109.23	39.56	125.35
	RT	14.35	1.09	110.51	39.75	126.63
	ST	14.96	1.15	113.37	42.33	129.94
	STS	15.83	1.22	121.74	46.23	133.42

### 2.2 Experimental design

The experiment was conducted from 2007 to 2022. Four tillage methods were set up in the field experiment, including perennial plowing tillage (PT), perennial rotary tillage (RT), perennial strip rotary tillage (ST), and subsoiling every two years combined with strip rotary tillage (STS) (subsoiling years were 2007, 2010, 2013, 2016, 2019, 2022). The operation procedures of different tillage practices are shown in **Table 2**.

**Table 2:** Operation procedures of different tillage practices.

Tillage	Operation procedure
Perennial rotary tillage (RT)	Returning maize straw (about 10, 000 kg ha^−1^) to the field → Base fertiliser spreading → Rotary cultivating two times with IGQN-200K-QY rotary cultivatora ^a^ (working depth was about 15 cm) → Harrowing two times → Forming the border-check→ Seeding with common seeder
Perennial plowing tillage (PT)	Returning maize straw (about 10, 000 kg ha^−1^) to the field → Base fertiliser spreading → Ploughing once with ILFQ330 share type plow^b^ (25 cm in depth) → Rotary cultivating two times with IGQN-200K-QY rotary cultivatora (working depth was about 15 cm) → Harrowing two times → Forming the border-check → Seeding with common seeder
Perennial strip rotary tillage (ST)	Returning maize straw (about 10, 000 kg ha^−1^) to the field → Completing rotary cultivation of sowing row (working depth was about 15 cm), base fertilizer application, forming border-check and seeding at the same time with the 2BMYF-10/5 multifunctional direct seeder in stubble^c^
Strip rotary tillage with a two-year subsoiling interval (STS)	Returning maize straw (about 10, 000 kg ha^−1^) to the field → Subsoiling once with the ZS-180 vibration subsoiler^d^ (working depth was about 38 cm) → Completing rotary cultivation of sowing row (working depth was about 15 cm), base fertilizer application, forming border-check and seeding at the same time with the 2BMYF-10/5 multifunctional direct seeder in stubble

^a^The manufacturers of IGQN-200K-QY rotary cultivator is YTO Group Corporation.

^b^The manufacturers of ILFQ330 share type plow is runlian Technology Development Co., Ltd.

^c^The manufacturers of 2BMYF-10/5 multifunctional planter is Yuncheng Gongli Co., Ltd.

^d^The manufacturers of ZS-180 vibration subsoiler is Yuncheng Gongli Co., Ltd.

The plot area was 160 m^2^ (4 m × 40 m), and each treatment was repeated three times. Before sowing, N 105 kg ha^-1^, P_2_O_5–_150 kg ha^-1^ and K_2_O 150 kg ha^-1^ were applied as base fertilizer, and N 135 kg ha^-1^ was applied at jointing stage. Apply N 240 kg ha^-1^ throughout the entire growth period (Zhang et al., 2025). Potassium sulfate, diammonium phosphate and urea was selected for potassium, phosphorus and nitrogen fertilizer respectively. Other field management measures were the same as high-yield fields.

Concurrently, a ^15^N micro plot experiment was set up in the field during the wheat growing season from 2022 to 2023. The ^15^N micro area was separated by iron frames with length, width and height of 45 cm, 15 cm and 30 cm respectively. Each treatment was provided with “^15^N urea base fertilizer+common urea topdressing” and “common urea base fertilizer+^15^N urea topdressing”. The micro area was made of iron frame, with an area of 15 cm × 45 cm, and the periphery was excavated vertically for 28 cm. The original structure of the soil was not damaged. The iron frame was sleeved into the soil to prevent the mixing of fertilizers inside and outside the frame. ^15^N urea was produced by Shanghai Research Institute of chemical industry, and its abundance was 10.21%. The specific application and sampling period of ^15^N are shown in **Table 3**.

**Table 3:** Application status and sampling period of ^15^N.

Iron frame number	Treatment	Sampling period
1	Base application 1.54 g ^15^N	Jointing
2	Apply 1.54 g of ^15^N at the base, and apply 1.98g of regular urea during the jointing stage	Anthesis
3	Apply 1.54 g of ^15^N at the base, and apply 1.98g of regular urea during the jointing stage	Maturity
4	Apply 1.54 g of regular urea as the base, followed by 1.98 g of ^15^N during the jointing stage	Anthesis
5	Apply 1.54 g of regular urea as the base, followed by 1.98 g of ^15^N during the jointing stage	Maturity

### 2.3 Soil sampling and analysis

During the 2022–2023 wheat growing season, soil samples of 0–30 cm soil layer were collected at the jointing stage, anthesis, mid grain filling stage and maturity stage. Every 15 cm was a layer and repeated three times. Three soil samples of the same soil depth were mixed into one sample and passed through a 2 mm sieve. Then each mixed sample was divided into two parts, one was stored at 4 °C and the other was dried for sample analysis.

#### 2.3.1 Soil nitrogen fractions

The soil samples were air dried, ground and passed through a 0.25 mm<sieve, and then analyzed for soil total N. The Kjeldahl N digestion procedure was used to analyze the total N concentration (Bao, 2000). Soil samples were extracted with 1 mol L^-1^ KCl, and NH_4_^+^-N and NO_3_^--^N were determined by continuous flow analyzer (AutoAnalyzer3-AA3, Seal Analytical, Germany) (Shi et al., 2022).

Particulate organic nitrogen (PON) was determined as follows: 20 g of air-dried soil was weighed and 60 mL of 5 g L^-1^ sodium hexametaphosphate solution was added. Then, it was dispersed at 90 r min^-1^ for 18 hours. The dispersed soil suspension was rinsed with distilled water on a 53 μm sieve until the water flow cleared. The soil on the sieve was transferred to an aluminum box and dried at 60 °C for 48 h. Determination of total nitrogen in PON was consistent with that in soil (Huang et al., 2022).

The soluble organic nitrogen (SON) content was determined by extracting 10 g of fresh soil with distilled water at a soil-to-water ratio of 2:1 (w/v) for 1 hour, followed by filtration.The total nitrogen content (mg kg^-1^) in the solution was determined by potassium persulfate oxidation method, and then the mineral nitrogen content (mg kg^-1^) in the solution was determined by flow analyzer. The soluble organic nitrogen content was the difference between the total nitrogen content and the mineral nitrogen content (Luo et al., 2015).

Soil microbial biomass nitrogen was determined by chloroform fumigation (Bao, 2000).

#### 2.3.2 Soil nitrogen transformation intensity

Soil N fixation intensity: 50 g of fresh soil that had been sieved through 2 mm was placed into a sterilized 100 mL beaker, and 1 g of glucose was added and stirred well, while soil moisture was kept at 60% of the water holding capacity in the field during incubation, and after incubation for 30 d in a constant temperature box at 25 °C, the soil was dried, sieved through 0.149 mm, and its total nitrogen content was determined, and the total N content of the uncultivated soil was used as a control. The calculate specific formulas according to Equations 1-4.

(1) $$Soil\;nitrogen\;fixation\;intensity\left(mg\;g^{-1}\right)=TN_{\text{cultured}}-TN_{\text{uncultured}}$$

Where TN_cultured_ and TN_uncultured_ are the total N content of cultured and uncultured soil samples (mg g^-1^), respectively.

Soil ammonification intensity: the soil incubation method was used (Lin et al., 2010), 10 g of soil sieved through 2 mm was put into sterilized 100 mL triangular flasks, and then 1 mL of 20 g L^-1^ peptone solution was added, and the water content of the soil was maintained at 60% of the maximum holding capacity during the incubation process, and then the soil samples were sealed and incubated in a thermostat at 25 °C for 7 d (Li Y. et al., 2024). In the ratio of water to soil of 5:1, 1 mol L^-1^ KCl solution was added to the cultured soil samples, and the soil samples were shaken for 1 h and filtered to determine the NH_4_^+^-N content of the cultured soil and the control soil, and the difference between the two was converted to the number of milligrams of NH_4_^+^-N per unit of dry soil.

(2) $$Soil\;ammonification\;intensity\left(mg\;g^{-1}\right)=C_{cultured}-B_{\text{uncultured}}$$

Where C_cultured_ and B_uncultured_ are NH_4_^+^-N content (mg g^-1^) of soil samples after culture and blank soil sample NH_4_^+^-N content (mg g^-1^).

Soil nitrification intensity: 10 g of dry soil sieved through 2 mm was taken into a 100 mL triangular flask, added with 2.5 mg N of ammonium sulfate solution, and incubated in a 28 °C thermostat, and taken out at 0, 3, 6, 9, 14, 21, and 28 d, respectively. 50 mL of 2 mol L^-1^ KCl solution was added, and filtered after shaking for 1 h. The nitrate nitrogen content was determined using a flow analyzer.

(3) $$Soil\;\;nitrification\;intensity\left(mg\;kg^{-1}\;d^{-1}\right)=C_{cultured}-N_{\text{uncultured}}$$

Where C_cultured_ and N_uncultured_ are the NO^3--^N content (mg kg^-1^ d^-1^) in days after culture and uncultured NO_3_^--^N content (mg kg^-1^ d^-1^).

Soil denitrification intensity: The nitrate disappearance method was used (Li et al., 2008). Weighing 10 g of soil through 2 mm sieve, placed in 25 mL triangular bottle, adding a certain amount of KNO_3_, so that the soil surface has a thin layer of submerged water. The triangular bottle was put into a vacuum desiccator, vacuumed, filled with nitrogen, and repeated several times to make the desiccator free of oxygen. Several milliliters of alkaline pyrogallic gallic acid were placed in the bottom of the desiccator to absorb the residual oxygen. The desiccator was incubated in a constant temperature room, and soil samples were taken out at 0 d, 1 d, 3 d, and 5 d. The residual nitrate nitrogen in the samples was measured.

(4) $$Soil\;denitrification\;intensity\left(mg\;kg^{-1}\;d^{-1}\right)=C_{cultured}-N_{\text{uncultured}}$$

Where C_cultured_ and N_uncultured_ are the NO_3_^--^N content (mg kg^-1^ d^-1^) in days after culture and uncultured NO_3_^--^N content (mg kg^-1^ d^-1^).

#### 2.3.3 Soil N-transforming enzyme activity

Soil N-transforming enzyme activity include urease, protease, nitrate reductase and nitrite reductase, and their activities were determined according to the method of Guan Songyin (Guan, 1986).

Determination of urease activity: 5 g of air-dried soil was taken and 1 mL of toluene was added. After 15 min, 10 mL of 10% urea solution and 20 mL of pH 6.7 citrate buffer were added. After shaking well, the soil was incubated at 37 °C for 24 h. After filtration, 3 mL of filtrate was injected into a 50 mL volumetric flask and then colorimetrically determined according to the method of colorimetric determination of the plotted standard curve.

Protease activity: 4 g of air-dried soil was weighed, 20 mL of 1% casein solution and 1mL of toluene were added, and placed in an incubator at 30 °C for 24h. At the end of the incubation period, 2 mL of 0.1N sulfuric acid and 12 mL of 20% sodium sulfate were added to the mixture to precipitate the proteins, then 2 mL of the supernatant was centrifuged for 15 min (6000 r min^-1^), placed in a 50 mL volumetric flask, and 1 mL of 2% ninhydrin was added to rinse the bottleneck. Then it was heated in boiling water bath for 10 min, diluted with distilled water to the scale, and colorimetric determination was carried out according to the color rendering method of drawing standard curves.

Nitrate reductase activity: 1 g fresh soil sample was taken in 100 mL plastic bottle, added with 20 mg CaCO_3_ and 1 mL KNO_3_ solution, mixed well and added with 1 mL glucose solution. The mixture was pumped for 3 min and incubated in a 30°C incubator for 24 h. After culture, 50 mL deionized water and 1 mL aluminum-potassium alum solution were added. Leave for 20 minutes. The mixture was mixed and filtered. 20 mL of the filtrate was dried on a porcelain evaporating dish, and dissolved in 2 mL phenol disulfonic acid solution for 10 min. Then 15 mL of deionized water was added, adjusted to a slightly yellow color with 10% NaOH, and finally transferred to a 50 mL volumetric bottle for colorimetric analysis at 400–500 mm after a fixed volume.

Nitrite reductase activity: 1 g of soil was added with 20 mg CaCO_3_, 1 mL of 0.5% NaNO_2_ was added, and 1 mL of 1% glucose solution was added after shaking. After that, the procedure was performed as for nitrate reductase. 1 mL of filtrate was taken into a 50 mL volumetric flask, 5 mL of water and 4 mL of Gerry reagent were added to mix, color was developed, fixed to 50 mL, and colorimetry was performed at 550 to 600 nm.

#### 2.3.4 NH_3_ volatilization and N_2_O emission

NH_3_ was collected by aeration (Wang et al., 2002). A rigid PVC tube of 15 cm diameter and 10 cm height was used. During NH_3_ collection, two sponges (each 2 cm thick and 16 cm in diameter) were uniformly immersed in 15 mL of glycerol phosphate solution (50 mL of phosphoric acid and 40 mL, for a total volume of 1000 mL) into the rigid PVC tube. The lower sponge for NH_3_ absorption was placed 4 cm from the bottom of the tube and the upper sponge for atmospheric isolation was placed flush with the top of the tube. For sampling, the lower sponge was removed and immediately placed in a sealed bag. Then, a new sponge saturated with glycerol phosphate solution was placed in the lower layer, and the upper layer was replaced every 3–7 days according to wet or dry conditions. The lower sponge was placed into 500 mL plastic bottle, and 300 mL 1.0 mol L^-1^ KCl solution was added. After shaking for 1 h, the content of NH_4_^+^-N was determined by continuous flow analyzer. Calculate specific formulas according to Equations 5-8.

Samples were taken once a day during the first week after fertiliser application; during the second to third weeks, samples were taken every 2 to 3 d, and the sampling interval was extended to 7 d thereafter until the rate of ammonia volatilisation was constant and sampling was stopped.

(5) $$F_{NH3}=\left[M/\left(A\cdot D\right)\right]/100$$

Where F_NH3_ is the NH_3_ volatilization rate (kg ha^-1^ d^-1^), and M is the average amount of ammonia (NH_3_-N, mg) measured each time by a single device in the aeration method; A is the cross-sectional area of the capture device (m^2^); D is the time of each successive capture (d).

The formula of cumulative ammonia volatilization is:

(6) $$CF_{NH3}=\frac{1}{2}\times \sum_{i=1}^{n}\left[(V_{i}+V_{i-1})\times (T_{i}-T_{i-1})\right]$$

Where CF_NH3_ is cumulative NH_3_ volatilization (kg ha^-1^), n is the total number of samples, V_i_ is the volatilization rate of the i sample of NH_3_, and T_i_ is the date of the i sample.

Soil N_2_O emission fluxes were measured by static chamber and gas chromatography (Agilent 7890A, Agilent Inc., USA) (Lyu et al., 2024). Sampling chambers were made of Plexiglas with frames of 50 cm, 50 cm and 100 cm in length, width and height, respectively. Each sampling chamber was matched with a frame base (50 cm length × 50 cm width × 20 cm height). Sampling was done continuously for a week after fertiliser application. Sampling was required after irrigation and precipitation. Gas samples were collected on each sampling day from 9:00 a.m. to 11:00 a.m. Gas samples were collected using a 50 mL syringe and the gas chamber was closed at 0, 10, 20, and 30 min after each sample, with four samples collected at 10-minute intervals and then transferred to an evacuated gas bag. Gas samples were analysed for N_2_O concentration within 48 h of collection using a gas chromatograph (Agilent 7890 A, Wilmington, USA) equipped with an electron capture detector (ECD) and a backwash unit controlled by a 10-port valve. The carrier gas consisted of 95% argon and 5% methane at a flow rate of 30 mL per minute. The oven and detector temperatures were set at 85 °C and 320 °C, respectively. A standard gas of 5 ppm was selected to establish the standard curve according to the concentration of the N_2_O sample. The analyses were performed using a manual injection technique. Specifically, 40 μL of sample was drawn up using a 100 μL closed syringe and the injection into the analyser was completed within 1 second.

(7) $$F=\rho \times \frac{V}{S}\times \frac{\Delta c}{\Delta t}\times \frac{273}{273+T}$$

Where F is the N_2_O flux (μg m^-2^ h^-1^); ρ is the density of N_2_O in the standardized state. V is the volume (m^3^) of the gas collection box; S is the soil area covered by the gas collection box (m^2^); Δc/Δt is the cumulative rate (ppm h^-1^); T is the temperature in the gas collection box (°C).

(8) $$N_{f}=\frac{1}{2}\times 24\times 10^{-5}\times \sum_{i=1}^{n}[(F_{i}+F_{i-1})\times \left(d_{i}-d_{i-1}\right)$$

Where N_f_ is the cumulative N_2_O emission (kg ha^-1^); I is the number of sampling times; D is the sampling date. The cumulative N_2_O emissions are calculated based on the emissions between adjacent measurement intervals.

#### 2.3.5 The utilization of nitrogen in wheat under ^15^N tracer technique

Determination of ^15^N: The total number of spikes in each microzone was investigated at the stage of jointing, anthesis and maturity, and all the plant samples in the microzone were taken and divided into samples according to each organ, killed at 105 °C for 30 min, and dried at 70 °C until constant weight. After taking the plant samples, soil samples were taken from 0–200 cm soil layer of each microzone by soil auger, with every 20 cm as a layer, mixed well and dried naturally. After drying, the samples were weighed to determine the dry matter accumulation. A high-throughput tissue grinder (GT200) was used for grinding, and the nitrogen content of each sample was determined by an Elementar vario MICRO cube elemental analyser (Elementar, Germany), and the ^15^N abundance value of each sample was determined by an Isoprime 100 mass spectrometer (Isoprime, UK) (Li et al., 2023) and calculated (Chen et al., 2019; Ye et al., 2022). See Equations 9-14 for specific calculation formula.

(9) Ndff(basal,topdressing%)=(a−b)/(c−d)×100

Where Ndff is the percentage of N obtained from nitrogen fertiliser, a is the ^15^N abundance of microzone plant samples, b is the ^15^N abundance of microzone samples treated with the same tillage treatment, c is the ^15^N abundance of ^15^N-labelled nitrogen, and d is the standard value of natural ^15^N abundance (0.3663% ^15^N).

The proportion (%) of nitrogen accumulated by plants from fertilizer nitrogen and soil nitrogen is calculated by the following formula:

(10) $$Ndff_{Total\;fertilizer}\left(\%\right)=Ndff_{basal}\left(\%\right)+Ndff_{topdressing}\left(\%\right)$$

(11) $$Ndff_{soil}\left(\%\right)=100-Ndff_{Total\;fertilizer}\left(\%\right)$$

The N accumulated by plants comes from the amount of fertilizer nitrogen (basal or topdressing nitrogen) and soil nitrogen (N kg ha^-1^), which is calculated by multiplying their respective ratios by the total plant nitrogen accumulation (PNA, kg ha^-1^).

The fate of fertiliser N included plant uptake, residues in the soil and losses from the wheat-soil system. Recovery of fertiliser N (%), residual fertiliser N (%) and loss of fertiliser N (%) were calculated using the following equations:

(12) $$Fertilizer\;N\;recovery\left(\%\right)=PNA_{fertilizer}/NA\times 100$$

(13) $$Fertilizer\;N\;residual\left(\%\right)=NRIS_{Total\;fertilizer}/NA\times 100$$

(14) Fertilizer N loss(%)=100−Fertilizer N recovery(%)−Fertilizer N residual(%)

Where PNA_fertilizer_, NRIS_Total fertilizer_ and NA represent plant fertiliser N accumulation (kg ha^-1^), fertiliser N residue (kg ha^-1^) and N application (kg ha^-1^), respectively.

### 2.4 Wheat yield and yield stability

During the 2018–2022 wheat growing season, the winter wheat yield of 3 m^2^ per cultivated plot was manually measured at maturity. Harvested kernels were dried in the sun and then yields were expressed as 12.5% grain moisture (Wu et al., 2023). Yield stability was expressed as the coefficient of variation (CV), which measures the degree of variability among treatments across years, with smaller values indicating higher yield stability. CV is the ratio of the standard deviation of yield (σ) to the mean yield (2018-2022) (Smith et al., 2007; Li et al., 2024).

### 2.5 Statistical analysis

Analysis of variance (ANOVA) tests were conducted to evaluate the direct and interactive effects of different tillage methods and soil depths on the measured variables. All data were statistically analyzed using SPSS 25.0 statistical package (IBM SPSS, Inc., Chicago, USA). The least significant difference method (LSD) was used to compare the mean value and interaction effectss, and the difference was determined to be significant at the probability level of P< 0.05. Pearson correlation coefficient was used to evaluate the relationship between soil N transformation intensity, soil N metabolism enzyme activity, soil N fractions and grain yield.

## 3 Results

### 3.1 Grain yield and yield stability

**Figure 3:**
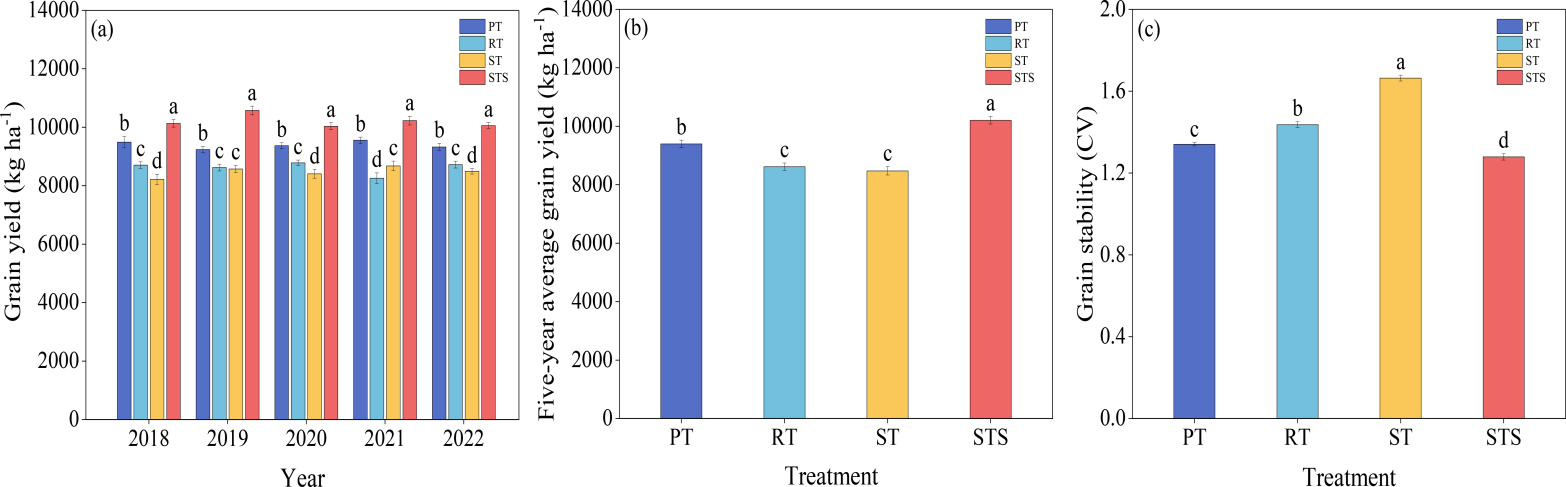
**(a)** Average grain yields of PT, RT and ST treatments from 2018 to 2022. **(b)** Average grain yield of PT, RT and ST treatments in five winter wheat growing seasons and **(c)** yield stability. Different lowercase letters indicate significant differences among different treatments (p< 0.05). The data are the mean ± standard deviation (SD) (n = 3). PT, perennial plowing tillage; RT, perennial rotary tillage; ST, perennial strip rotary tillage; STS, subsoiling every two years combined with strip rotary tillage.

Across five wheat growing seasons, STS had higher grain yield than PT, RT and ST treatments (**Figure 3A**). The five-year average yield of STS treatment increased by 8.62%, 18.42% and 20.44% respectively compared with PT, RT and ST treatments (**Figure 3B**). In addition, the coefficient of variation (CV) of STS treatment was significantly lower than that of other treatments (**Figure 3C**). The smaller the CV, the better the yield stability. This indicated that STS treatment had better yield stability.

### 3.2 Soil nitrogen transformation process

#### 3.2.1 Soil nitrogen transformation intensity

**Figure 4:**
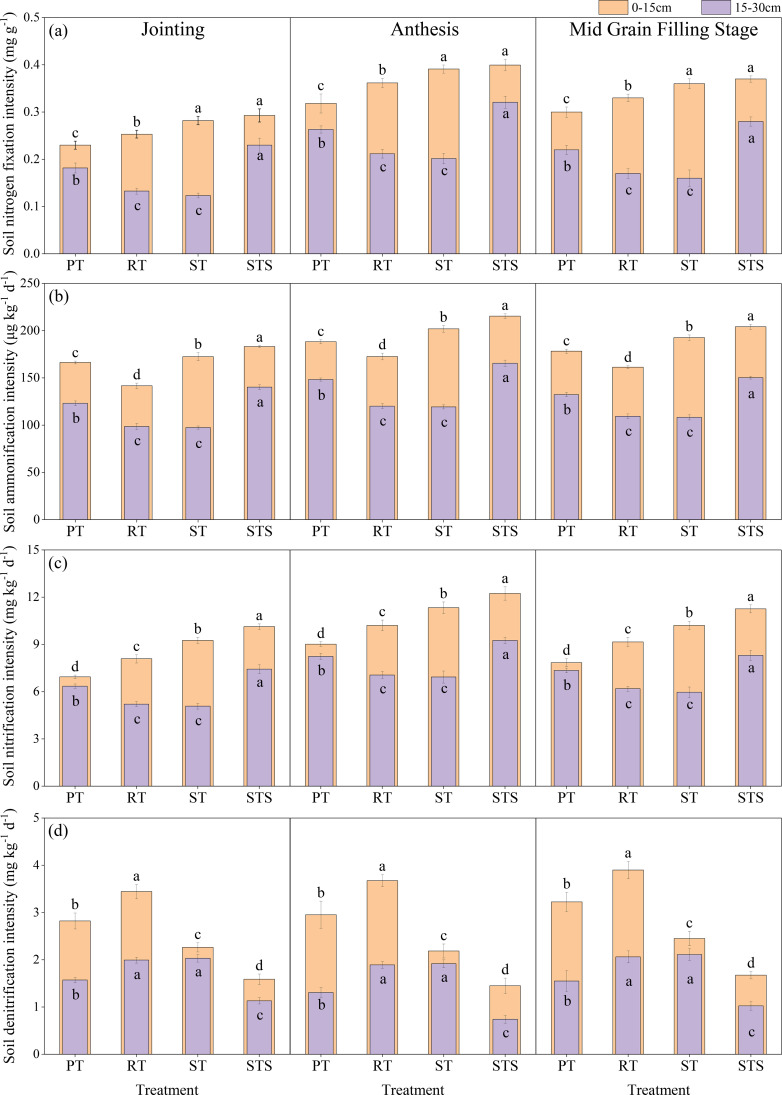
soil nitrogen fixation intensity (SNFI, **(a)**), ammonification intensity (SAI, **(b)**), nitrification intensity (SNI, **(c)**) and denitrification intensity (SDI, **(d)**) after different tillage treatments at jointing, anthesis and mid grain filling stages. Different lowercase letters indicate significant differences among different treatments (p< 0.05). The data are the mean ± standard deviation (SD) (n = 3). PT, perennial plowing tillage; RT, perennial rotary tillage; ST, perennial strip rotary tillage; STS, subsoiling every two years combined with strip rotary tillage.

The tillage method significantly affects the intensity of soil nitrogen transformation (**Figure 4**). The intensity of soil nitrogen transformation is significantly affected by tillage, soil depth and their interactions (**Table 4**). As the growth period progresses, the SNFI, SAI and SNI under different tillage methods first increased and then decreased, reaching the maximum value at anthesis (**Figures 4A–C**), while the SDI showed the opposite trend (**Figure 4D**). The SNFI, SAI, SNI and SDI decreased with the increase of soil depth (**Figure 4**). In the 0–15 cm soil layer, STS significantly enhanced the SNFI, SAI and SNI at different growth stages of wheat compared with other treatments (**Figures 4A–C**), but significantly reduced the SDI (**Figure 4D**). In the 15–30 cm soil layer, the SNFI, SAI and SNI were the highest in the STS treatment, followed by PT, and then ST and RT, with no significant difference between the latter two, while the SDI was significantly higher in RT and ST than in PT treatment, and the lowest in STS treatment (**Figure 4**).

**Table 4:** Significance levels of soil nitrogen transformation intensity and N-transforming enzyme activity in a two-way ANOVA of tillage practices and soil depth.

Factors	SNFI (mg g^-1^)	SAI (μg kg^-1^ d^-1^)	SNI (mg kg^-1^ d^-1^)	SDI (mg kg^-1^ d^-1^)	UR (mg g^-1^ d^-1^)	PR (mg g^-1^ d^-1^)	NR (mg g^-1^ d^-1^)	NRE (mg g^-1^ d^-1^)
Tillage (T)								
PT	0.25b	156.16b	7.62c	2.01b	0.95b	1.01b	0.19b	0.40b
RT	0.24b	133.98d	7.65c	2.44a	0.86c	0.94c	0.22a	0.44a
ST	0.25b	148.71c	8.13b	1.87c	0.90c	0.98bc	0.19b	0.41b
STS	0.32a	176.50a	9.77a	1.11d	1.06a	1.09a	0.15c	0.35c
Soil depth (D)								
0–15 cm	0.32a	181.55a	9.64a	2.32a	1.05a	1.13a	0.21a	0.43a
15–30 cm	0.21b	126.12b	6.95b	1.40b	0.83b	0.88b	0.17b	0.37b
ANOVA								
T	***	***	***	***	***	***	***	***
D	***	***	***	***	***	***	***	***
T×D	***	***	***	***	***	***	***	***

SNFI, soil nitrogen fixation intensity; SAI, ammonification intensity; SNI, nitrification intensity; SDI, denitrification intensity; UR, soil urease; PR, protease; NR, nitrate reductase; NRE, nitrite reductase. PT, perennial plowing tillage; RT, perennial rotary tillage; ST, perennial strip rotary tillage; STS, subsoiling every two years combined with strip rotary tillage. Different letters within a column indicate the significant differences for tillage treatments at the level of P< 0.05 (LSD test). *, ** and *** correspond to P<0.05, P<0.01 and P<0.001, respectively.

#### 3.2.2 Soil N-transforming enzyme activity

**Figure 5:**
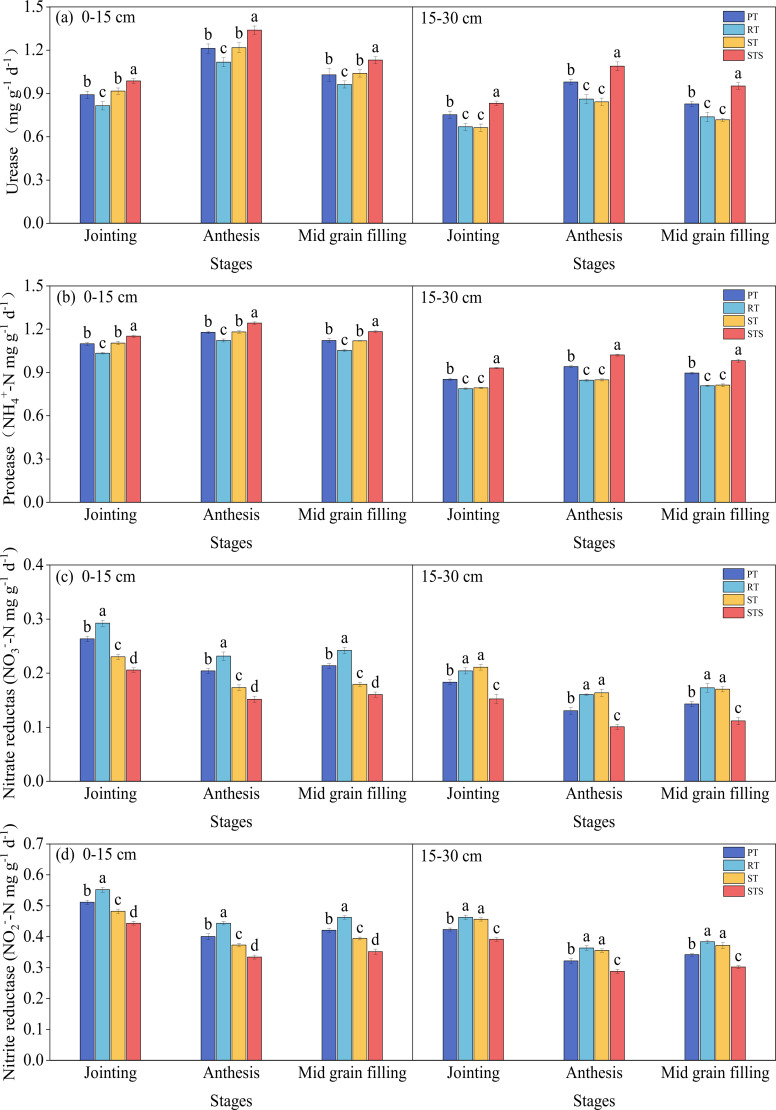
Activities of soil urease (UR, **(a)**), protease (PR, **(b)**), nitrate reductase (NR, **(c)**), and nitrite reductase (NRE, **(d)**) after different tillage treatments at the middle stages of jointing, anthesis, and mid grain filling. Different lowercase letters indicate significant differences among different treatments (p< 0.05). The data are the mean ± standard deviation (SD) (n = 3). PT, perennial plowing tillage; RT, perennial rotary tillage; ST, perennial strip rotary tillage; STS, subsoiling every two years combined with strip rotary tillage.

The urease and protease activities increased from jointing stage to anthesis, and then decreased to the middle grain filling stage (**Figures 5A, B**). The nitrate reductase and nitrite reductase activities decreased from jointing stage to anthesis, but increased in the mid grain filling stage (**Figures 5C, D**). The activity of soil N-transforming enzymes was significantly affected by tillage, soil depth and their interactions (**Table 4**). The soil N-transforming enzyme activity in the 0–15 cm soil layer were higher than those in the 15–30 cm soil layer (**Figure 5**). In the 0–15 cm soil layer, compared with PT, RT and ST, STS treatment had the highest urease and protease activities at jointing, anthesis and mid grain filling stage, followed by ST and PT treatments, and RT was the lowest. In the 15–30 cm soil layer, STS was significantly higher than other treatments (**Figures 5A, B**). However, STS treatment significantly reduced nitrate reductase and nitrite reductase activities in the 0–30 cm soil layer at different growth stages (**Figures 5C, D**).

#### 3.2.3 NH_3_ volatilization and N_2_O emission

**Figure 6:**
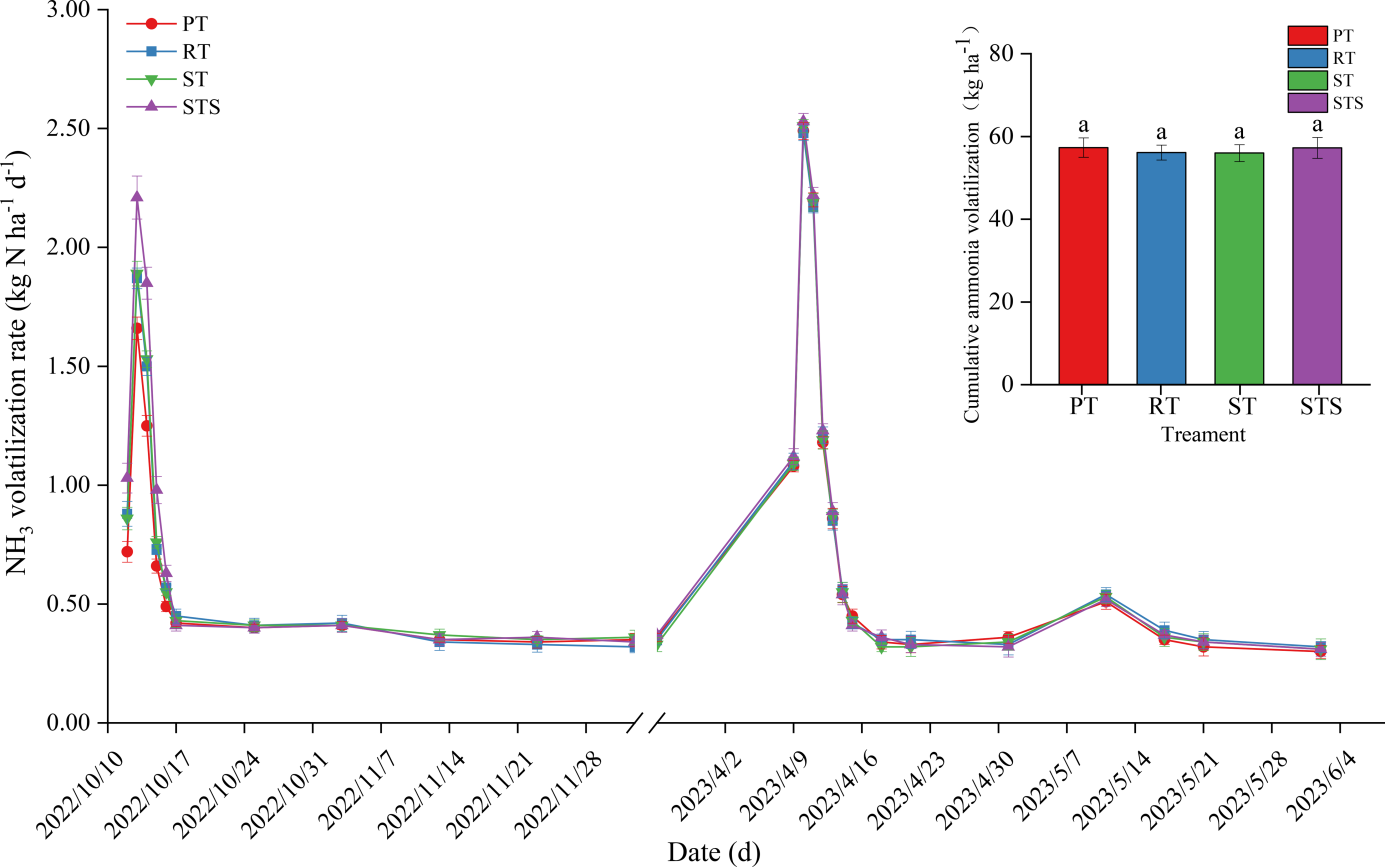
NH_3_ volatilization rate and cumulative NH_3_ volatilization under different tillage methods during the whole growth period of winter wheat. Different lowercase letters indicate significant differences among different treatments (p< 0.05). The data are the mean ± standard deviation (SD) (n = 3). PT, perennial plowing tillage; RT, perennial rotary tillage; ST, perennial strip rotary tillage; STS, subsoiling every two years combined with strip rotary tillage.

All treatments peaked on the second day after fertilization, and then gradually leveled off until there was no significant difference between the treatments. The maximum ammonia volatilization rates after basal and topdressing were 2.21 and 2.53 kg N ha^-1^ d^-1^, respectively. The ammonia volatilization rate was significantly increased by irrigation on May 11, and then stabilized (**Figure 6**). The ammonia volatilization rate of STS treatment was significantly higher than that of other treatments during the sowing period. After that, there was no significant difference in the ammonia volatilization rate among the treatments in each period, which made the cumulative ammonia volatilization in the whole growth period showed no significant difference among the treatments (**Figure 6**).

**Figure 7:**
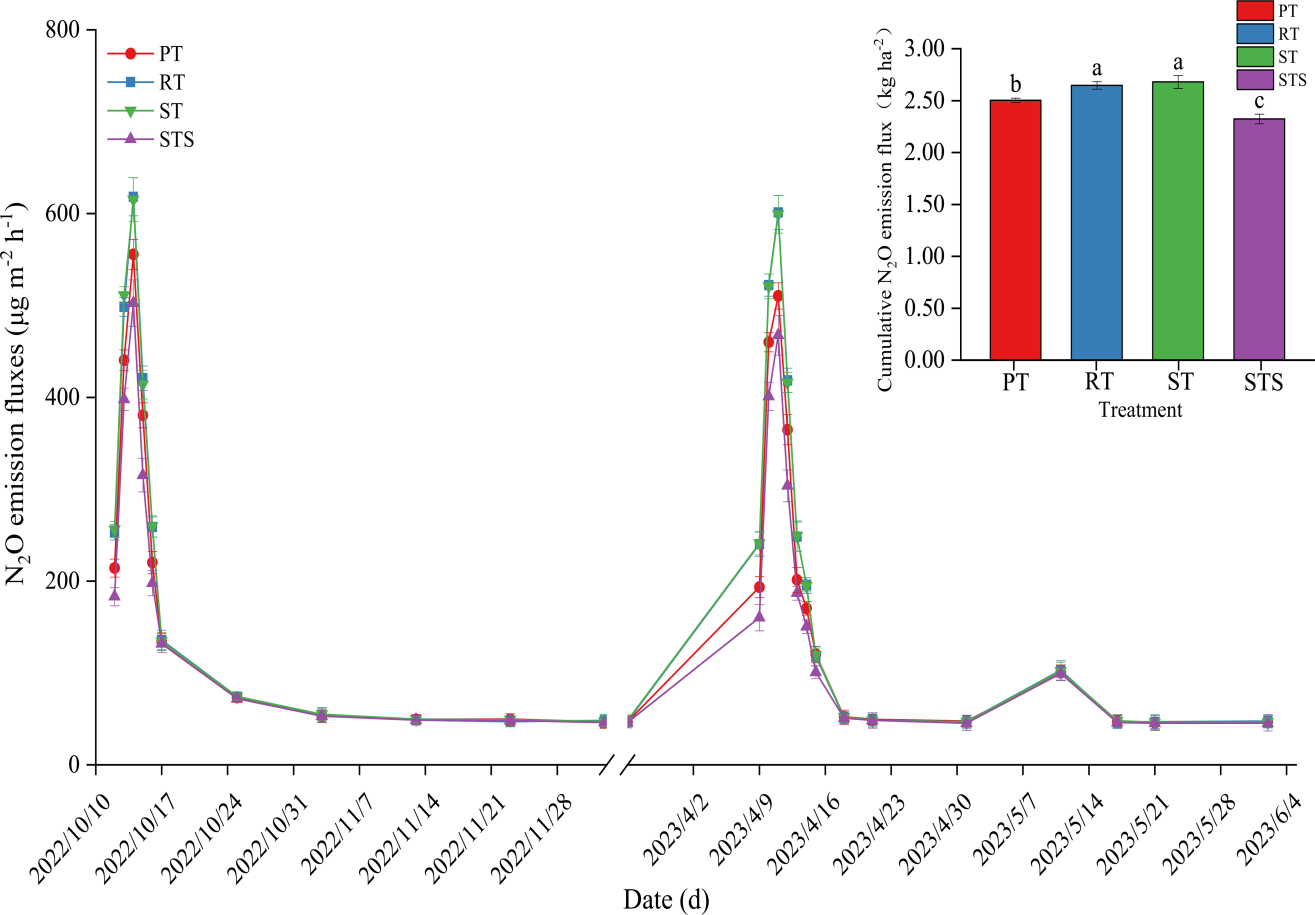
N_2_O volatilization rate and cumulative N_2_O emission under different tillage methods during the whole growth period of winter wheat. Different lowercase letters indicate significant differences among different treatments (p< 0.05). The data are the mean ± standard deviation (SD) (n = 3). PT, perennial plowing tillage; RT, perennial rotary tillage; ST, perennial strip rotary tillage; STS, subsoiling every two years combined with strip rotary tillage.

The peak value of N_2_O emission flux mainly occurred in a period of time after base fertilizer application and topdressing, reaching the maximum value at 3 days after fertilization, and then stabilized until there was no significant difference between the treatments. After irrigation on May 11, the N_2_O emission flux increased significantly and then stabilized (**Figure 7**). The cumulative volatilization of N_2_O was the highest in RT and ST treatments, and there was no significant difference between them, followed by PT treatment, and STS treatment was the lowest. The cumulative N_2_O emission flux of STS treatment was 7.19%, 12.22% and 13.31% lower than that of PT, RT and ST, respectively (**Figure 7**).

### 3.3 Soil nitrogen fractions

**Figure 8:**
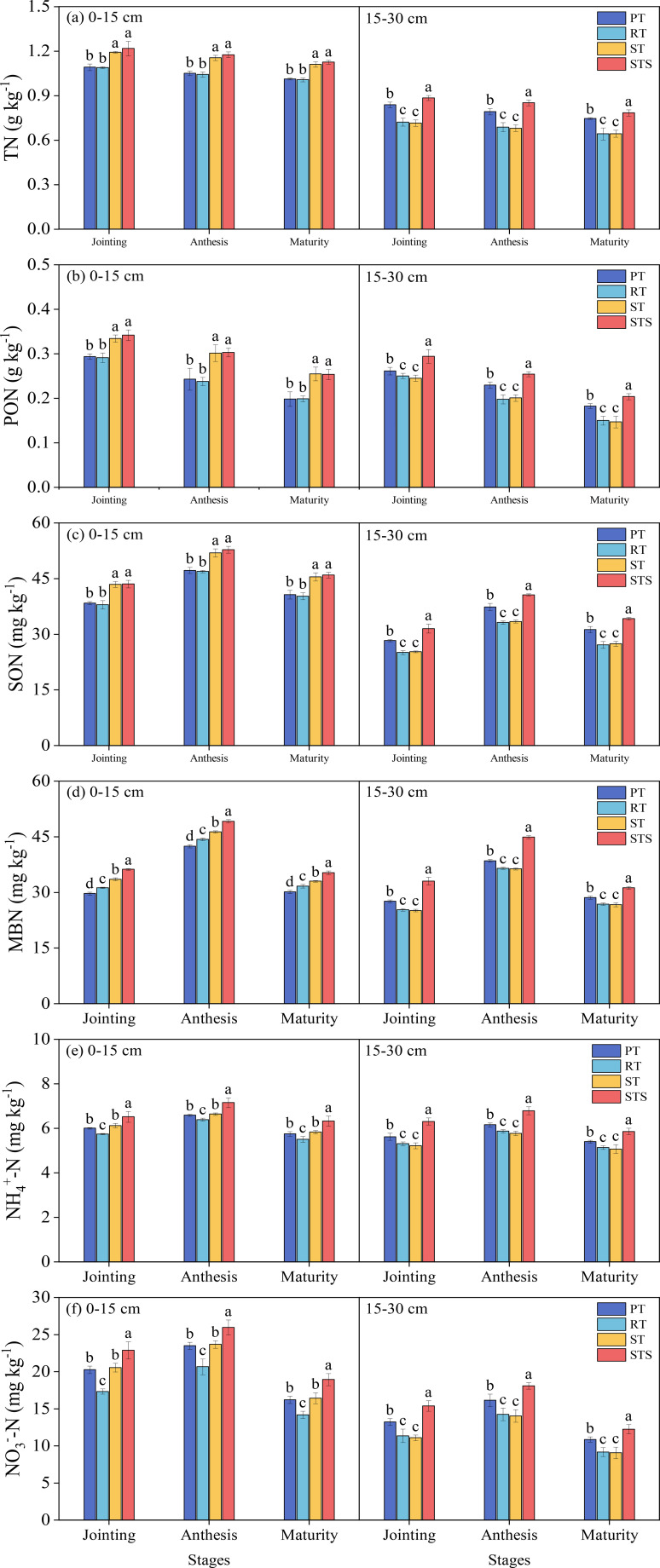
The contents of total nitrogen (TN, **(a)**), particulate organic nitrogen (PON, **(b)**), soluble organic nitrogen (SON, **(c)**), microbial biomass nitrogen (MBN, **(d)**), ammonium nitrogen (NH_4_^+^-N, **(e)**), and nitrate nitrogen (NO_3_^--^N, **(f)**) in the soil after different tillage treatments at jointing, flowering, and maturity stages. Different lowercase letters indicate significant differences among different treatments (p< 0.05). The data are the mean ± standard deviation (SD) (n = 3). PT, perennial plowing tillage; RT, perennial rotary tillage; ST, perennial strip rotary tillage; STS, subsoiling every two years combined with strip rotary tillage.

The TN and PON contents in 0–30 cm soil layer under different tillage methods showed a decreasing trend from jointing stage to maturity stage (**Figures 8A, B**). However, the SON, MBN, NH_4_^+^-N and NO_3_^--^N contents in 0–30 cm soil layer increased first and then decreased with the growth process, and reached the maximum at anthesis (**Figures 8C–F**). The TN, PON, SON, MBN, NH_4_^+^-N and NO_3_^--^N contents decreased with the deepening of soil layer (**Figure 8**). The contents of TN, PON and SON in 0–15 cm soil layer of STS and ST were significantly higher than those of PT and RT treatments. The MBN, NH_4_^+^-N and NO_3_^--^N contents in STS were significantly higher than those in other treatments (**Figure 8**). In the 15–30 cm soil layer, the TN, PON, SON, MBN, NH_4_^+^-N and NO_3_^--^N contents in STS were significantly higher than those in PT, RT and ST treatments (**Figure 8**). Tillage practices, soil depth, and their interactions significantly affected soil N fraction content (**Table 5**).

**Table 5:** Significance levels of soil nitrogen fractions in a two-way ANOVA of tillage practices and soil depth.

Factors	TN (g kg^-1^)	PON (g kg^-1^)	SON (mg kg^-1^)	MBN (mg kg^-1^)	NO_3_^--^N (mg kg^-1^)	NH_4_^+^-N (mg kg^-1^)
Tillage (T)						
PT	0.92b	0.23c	37.20b	32.86c	16.71b	5.92b
RT	0.87c	0.22d	35.09c	32.66c	14.50d	5.66b
ST	0.92b	0.25b	37.85b	33.51b	15.81c	5.78b
STS	1.01a	0.28a	41.45a	38.33a	18.92a	6.49a
Soil depth (D)						
0–15 cm	1.11a	0.27a	44.56a	36.94a	20.06a	6.22a
15–30 cm	0.75b	0.22b	31.24b	31.74b	12.91b	5.71b
ANOVA						
T	***	***	***	***	***	***
D	***	*	***	***	**	***
T×D	***	***	***	***	***	***

TN, total nitrogen; PON, particulate organic nitrogen; SON, soluble organic nitrogen; MBN, microbial biomass nitrogen; NH_4_^+^-N, ammonium nitrogen; NO_3_^--^N; nitrate nitrogen. PT, perennial plowing tillage; RT, perennial rotary tillage; ST, perennial strip rotary tillage; STS, subsoiling every two years combined with strip rotary tillage. Different letters within a column indicate the significant differences for tillage treatments at the level of P< 0.05 (LSD test). *, ** and *** correspond to P<0.05, P<0.01 and P<0.001, respectively.

### 3.4 The utilization of nitrogen in wheat under ^15^N tracer technique

**Figure 9:**
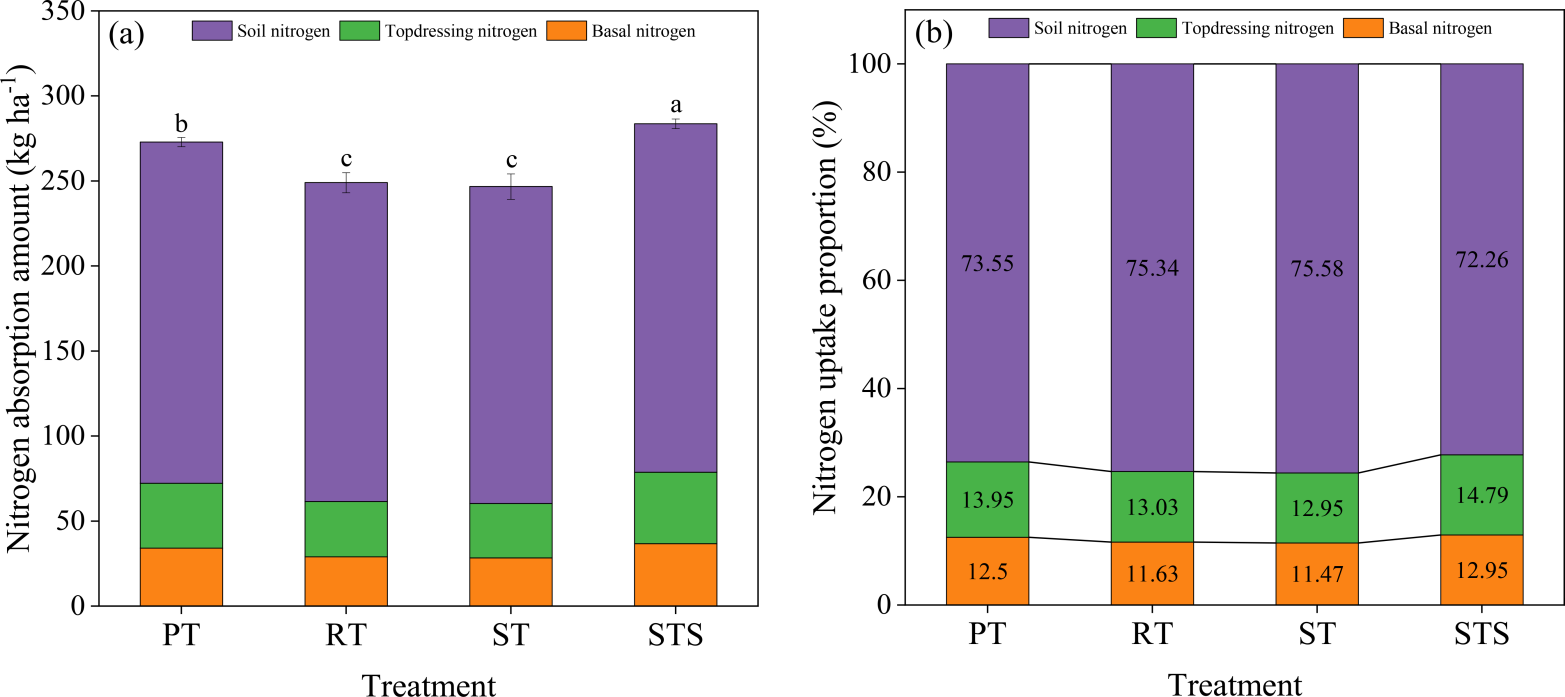
Absorption of nitrogen from different sources in wheat plants under different tillage treatments. **(a)** The absorption of nitrogen from different sources by wheat plants. **(b)** The absorption ratio of nitrogen from different sources in wheat plants. Different lowercase letters indicate significant differences among different treatments (p< 0.05). The data are the mean ± standard deviation (SD) (n = 3). PT, perennial plowing tillage; RT, perennial rotary tillage; ST, perennial strip rotary tillage; STS, subsoiling every two years combined with strip rotary tillage.

Among the different sources of N absorption in wheat, wheat had the highest amount and proportion of soil N absorption, followed by topdressing nitrogen, and basal nitrogen was the least (**Figure 9**). Tillage methods significantly affected the absorption of N by plants. The N uptake of wheat in soil and fertilizer by STS treatment was the largest, followed by PT, while RT and ST treatments were significantly lower than PT, and the total N uptake of STS was 4.0%, 13.91% and 14.98% higher than PT, RT and ST, respectively (**Figure 9A**).

**Figure 10:**
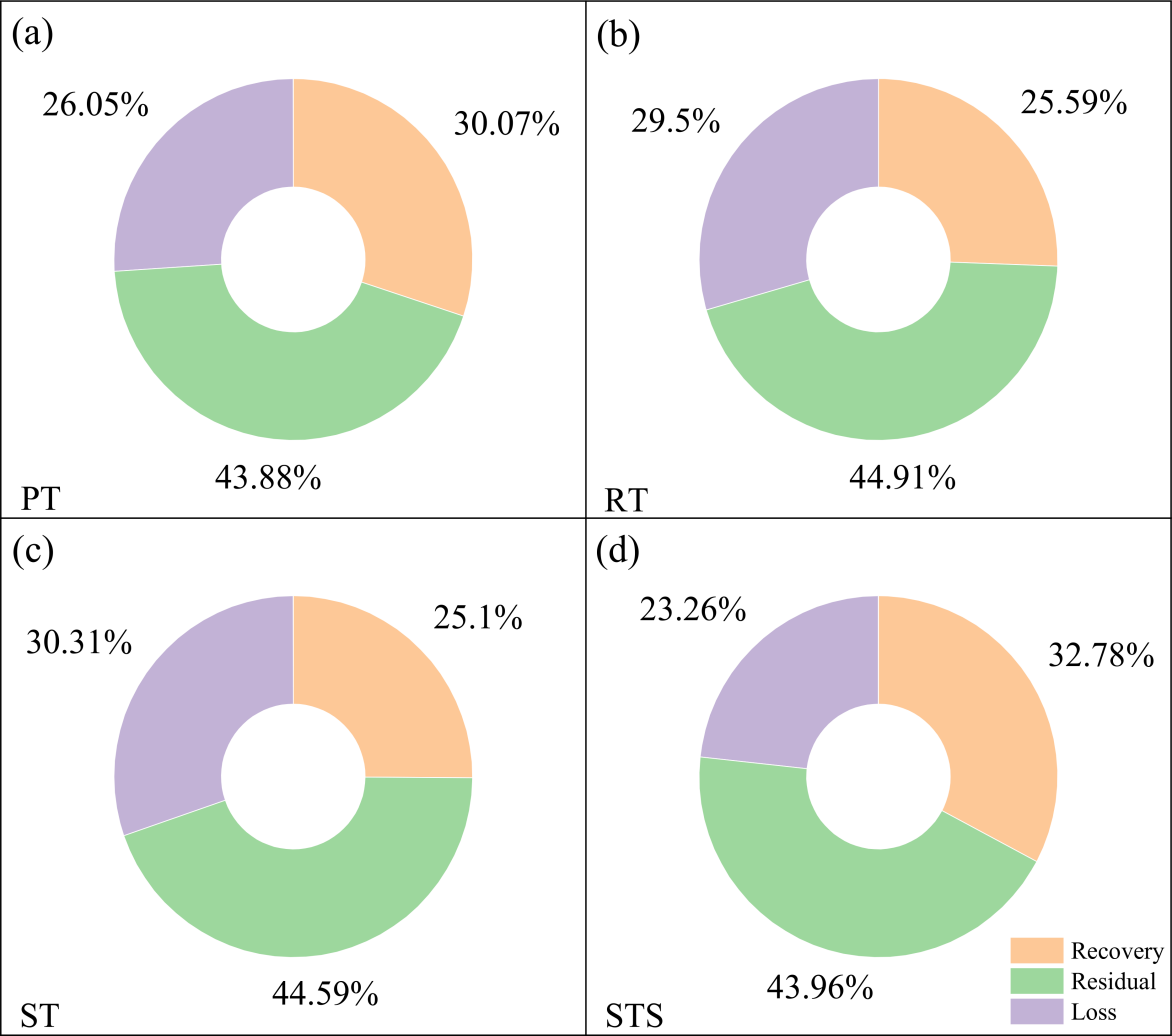
The fate of ^15^N fertilizer after different tillage treatments. The figures show the fate of 15N fertilizer after treatment with **(a)** PT, **(b)** RT, **(c)** ST, and **(d)** STS. PT, perennial plowing tillage; RT, perennial rotary tillage; ST, perennial strip rotary tillage; STS, subsoiling every two years combined with strip rotary tillage.

Analysis of ^15^N fate revealed no significant differences in the proportion of N fertilizer residues in the soil of each tillage treatment (**Figure 10**). STS treatment significantly increased the proportion of N fertilizer absorbed by wheat, which was 9.01%-30.6% higher than other treatments. At the same time, STS treatment significantly reduced the proportion of N fertilizer loss, which was 10.71%, 26.83% and 30.31% lower than PT, RT and ST treatments, respectively (**Figure 10**).

### 3.5 Relationships between soil nitrogen transformation ability, soil nitrogen supply ability and crop yield

**Figure 11:**
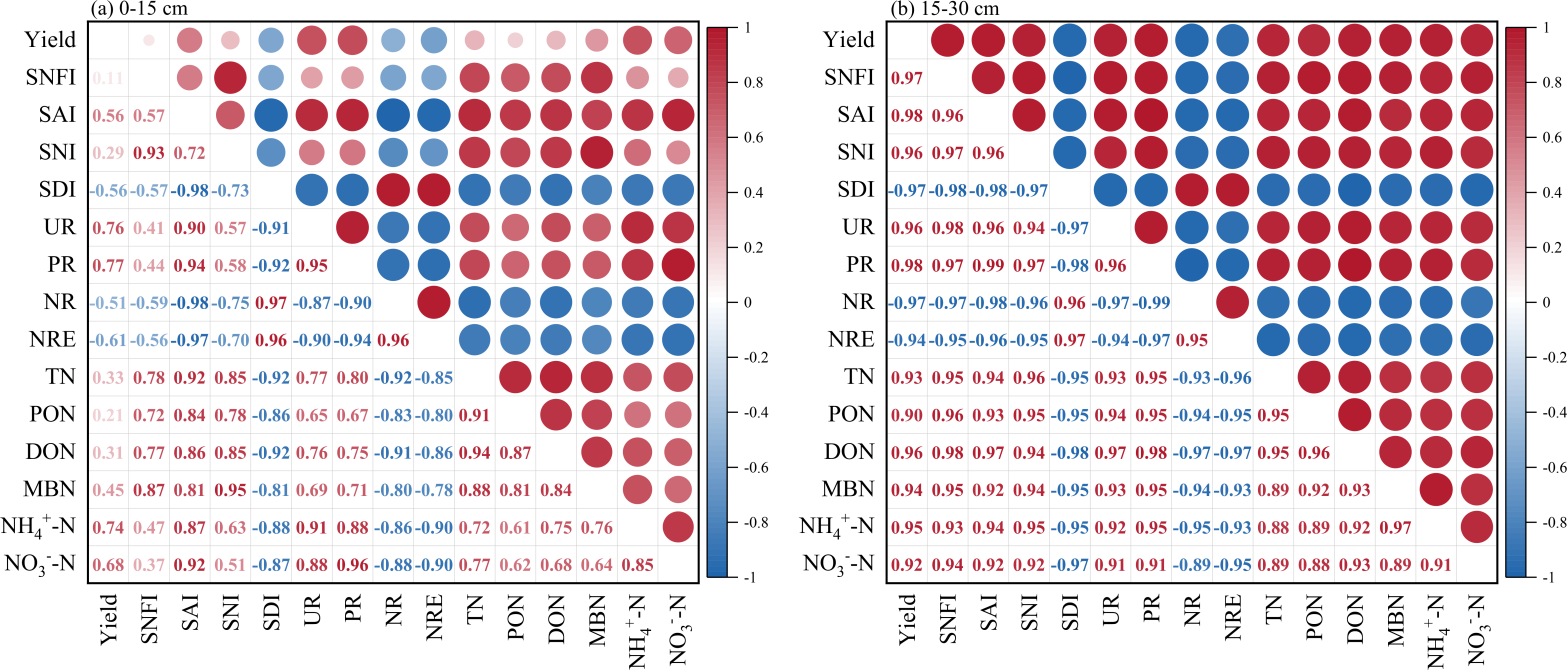
Correlation between average yield of winter wheat at maturity stage (June 11, 2023) and soil nitrogen transformation intensity, soil nitrogen invertase activity and soil nitrogen fractions at soil depths of 0-15 cm **(a)** and 15-30 cm **(b)** during flowering stage. Yield, grain yield; SNFI, soil nitrogen fixation intensity; SAI, soil ammonification intensity; SNI, soil nitrification intensity; SDI, soil denitrification intensity; UR, urease; PR, protease; NR, nitrate reductase; NRE, nitrite reductase; TN, total nitrogen; PON, particulate organic nitrogen; DON, soluble organic nitrogen; MBN, microbial biomass nitrogen; NH_4_^+^-N, ammonium nitrogen; NO_3_^--^N, nitrate nitrogen.

In the 0–15 cm soil layer, wheat yield was positively correlated with SAI, SNI, UR, PR, and soil N fractions, while wheat yield was negatively correlated with SDI, NR and NRE activity (**Figure 11A**). In the 15–30 cm soil layer, wheat yield was positively correlated with SNFI, SAI, SNI, UR, PR, and soil N fractions, and negatively correlated with SDI, NR and NRE activity (**Figure 11B**).

**Figure 12:**
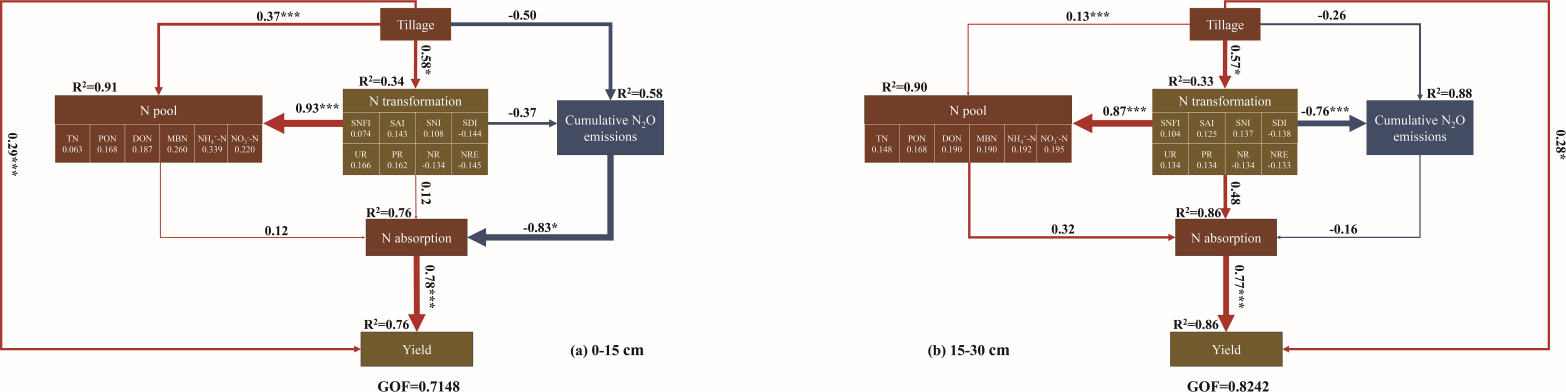
Partial Least Squares Path Modeling (PLS-PM) revealed the effects of different tillage practices, N transformation, N fractions, cumulative N₂O emissions, N uptake, and wheat yield in the 0-15 cm **(a)** and 15-30 cm **(b)** soil layers. Values above and below arrows represent standardized path coefficients and significance levels. Red and blue arrows indicate positive and negative effects, respectively. * P< 0.05; ** P< 0.01; *** P< 0.001. Yield, grain yield; SNFI, soil nitrogen fixation intensity; SAI, soil ammonification intensity; SNI, soil nitrification intensity; SDI, soil denitrification intensity; UR, urease; PR, protease; NR, nitrate reductase; NRE, nitrite reductase; TN, total nitrogen; PON, particulate organic nitrogen; DON, soluble organic nitrogen; MBN, microbial biomass nitrogen; NH_4_^+^-N, ammonium nitrogen; NO_3_^--^N, nitrate nitrogen.

Partial Least Squares Path Modeling (PLS-PM) revealed that path tillage affects N uptake and wheat yield by influencing N conversion, N pool composition, and cumulative N_2_O emissions (**Figure 12**). In the 0–15 cm soil layer, tillage practice significantly and positively influenced N mineralization (standard path coefficient = 0.58, P< 0.05) and N pool composition (standard path coefficient = 0.37, P< 0.001), while exerting a negative effect on cumulative N_2_O emissions. N transformation exerted a positive influence on the N pool (standard path coefficient = 0.93, P< 0.001), while the N pool positively affected N uptake. Conversely, cumulative N_2_O emissions significantly negatively impacted N uptake (standard path coefficient = -0.83, P< 0.001). N uptake significantly positively influenced yield (standardized path coefficient = 0.78, P< 0.001). Furthermore, tillage practice directly positively influenced yield (standardized path coefficient = 0.29, P< 0.001). At the 15–30 cm soil layer, tillage practices also significantly and positively influenced N transformation (standard path coefficient = 0.57, P< 0.05) and N pool composition (standard path coefficient = 0.13, P< 0.001). N transformation exerted a significant positive effect on the N pool (standardized path coefficient = 0.87, P< 0.001) and a significant negative effect on cumulative N_2_O emissions (standardized path coefficient = -0.76, P< 0.001). Both N transformation and N pool positively influenced N uptake, whereas cumulative N_2_O emissions negatively affected N uptake. N uptake significantly and positively influenced yield (standardized path coefficient = 0.77, P< 0.001). Additionally, tillage significantly and positively influenced yield (standardized path coefficient = 0.28, P< 0.05).

## 4 Discussion

### 4.1 Effects of tillage methods on wheat yield and yield stability

The grain yield of wheat is significantly affected by tillage methods (Yousefian et al., 2021). Under the condition of straw returning, the yield of no-tillage is 18.6%-27.3% higher than that of traditional tillage (Wen et al., 2023). However, some studies have suggested that no-tillage could reduce the yield of wheat, especially for many years, no-tillage often leads to a decrease in yield. This may be that long-term no-tillage leads to shallower plough layer and thicker plough layer, which limits the extension of roots and is not conducive to the absorption of deep nutrients and water by wheat (Wang et al., 2020). Similar findings were found in this study. After 16 years of long-term tillage, strip rotary tillage significantly reduced yield. However, on the basis of strip rotary tillage, the yield of STS (subsoiling every two years) remained at a high level compared with PT, RT and ST (**Figure 3A**). According to the average yield of wheat in 5 years, STS significantly increased wheat yield, which increased by 8.62%-20.44% compared with other treatments (**Figure 3B**). It may be that subsoiling can break the plow pan, promote the root system, and enhance the utilization of soil moisture and nutrients by wheat, thereby increasing the yield of wheat (Li et al., 2021). More importantly, the yield increasing advantage of STS is closely related to the increased N uptake by plants. This study found through ^15^N that STS significantly promotes wheat absorption of N from base and topdressing fertilizers, increases plant N accumulation, and reduces fertilizer N loss (**Figures 9**, **10**). This indicates that STS can meet the nitrogen requirements of wheat, thereby increasing yield. In addition, STS treatment had a lower coefficient of variation (**Figure 3C**), indicating that STS had better yield stability and was a sustainable and effective tillage mode.

### 4.2 Effects of tillage methods on soil nitrogen transformation process

In this study, the nitrogen fixation, ammonification and nitrification of soil under various tillage methods showed great differences at different growth stages, and reached the maximum at anthesis (**Figures 4A–C**). This might be because the anthesis of wheat is the peak period of vegetative growth and reproductive growth, the root amount reaches the maximum, and the microbial activity is the highest, which accelerates the N transformation process of soil. The soil denitrification intensity showed the opposite trend, which was the lowest at anthesis (**Figure 4D**). Due to the absorption and utilization of nitrogen by wheat at anthesis, the amount of N-containing compounds such as nitrate required for denitrification was reduced, thereby reducing the soil denitrification. Increasing soil disturbance can promote soil ammonification (Butnan and Vityakon, 2020), while no-tillage could inhibit ammonification and increase nitrogen fixation in the soil due to reducing soil disturbance, which will affect the absorption of N by crops during the N demand period, thereby affecting crop development (Nevins et al., 2021). In this study, the SNFI, SAI and SNI of STS in 0–30 cm soil layer at different growth stages were higher than those of PT, RT and ST. It may be that deep loosening makes root growth vigorous, and the oxygen concentration in deep soil layer is high, which promotes the improvement of microbial activity of N conversion. At the same time, the developed root system of STS treatment absorbs more N, reduces the substrate of denitrification, and further reduces the denitrification intensity, which can meet the demand of plant growth for N.

The deep soil layer caused by no-tillage conditions has low nutrient availability and microbial activity, which may reduce the N-transforming enzyme activity in the deep soil layer (Su et al., 2024). In this study, soil N-transforming enzyme activity also decreased with soil depth after tillage treatment (**Figure 5**), which was consistent with previous research results (Wu et al., 2021). The reason for the difference in soil N-transforming enzyme activity may be related to the number of substrates at different soil depths. Compared with PT, RT and ST, STS treatment significantly increased the activities of urease and protease in 0–30 cm soil layer (**Figures 5A, B**), but significantly decreased the activities of NR and NRE (**Figures 5C, D**). On the one hand, compared with other treatments, subsoiling increases the permeability of the deep soil layer while reducing soil disturbance, enhances the water storage capacity of the soil, and makes the hydrothermal conditions of different soil layers more suitable for the survival of microorganisms (Ning et al., 2022). On the other hand, the roots of deep tillage crops are developed, especially in the anthesis, the root exudates and residues are more(Zhang et al., 2023; Zhang et al., 2022), which greatly improves the activity of soil urease and protease, and makes the activity of soil urease and protease in anthesis reach the maximum. STS-treated wheat had a large demand for nutrients during the rapid growth period, reducing the reaction substrates of nitrate reductase and nitrite reductase, thereby reducing the activity of soil nitrate reductase and nitrite reductase. We also found that the intensity of soil ammonification and nitrification were positively correlated with urease and protease (**Figure 11**), indicating that the increase in N-transforming enzyme activity has a positive impact on soil N transformation process during long-term cultivation.

In addition, soil N transformation is closely related to N loss in soil systems (He et al., 2023). Globally, only 50% of fertilizer N is converted, and the rest is lost in different forms (Mălinaş et al., 2022). Among them, most of the active N lost from crop fields enters the atmosphere in the form of NH_3_ and N_2_O, and is affected by the soil N transformation process (Deng et al., 2023). We found that tillage methods significantly affected the ammonia volatilization rate after the application of base fertilizer nitrogen, especially the ammonia volatilization rate of STS treatment was significantly higher than that of PT, RT and ST. After STS treatment, the activity of soil urease and protease was enhanced, which promoted soil ammonification and increased the rate of ammonia volatilization (Wang et al., 2020). At the same time, due to the small soil disturbance of subsoiling, the fertilizer was concentrated in the surface layer, which reduced the contact between NH_4_^+^ and soil clay and organic matter, reduced the opportunity of adsorption, and then increased the volatilization of NH_3_ (Ma et al., 2021). However, there was no significant difference in the cumulative ammonia volatilization of each treatment. Perhaps it is because subsoiling root growth and enhances the absorption and assimilation of NH_4_^+^- N by plants. On the other hand, subsoiling improved soil aeration, accelerated the conversion of NH_4_^+^- N to NO_3_^--^ N, enhanced nitrification (**Figure 4C**), reduced the substrate for ammonia volatilization, resulting in a decrease in the duration and total amount of ammonia volatilization, with no difference in cumulative ammonia volatilization. As a key microbial anaerobic process, denitrification will produce gaseous loss in the form of N_2_O (Al-Hazmi et al., 2022). Tillage measures mainly affect N_2_O emissions by changing soil moisture, carbon source and oxygen availability (Pareja-Sánchez et al., 2020). Conservation tillage can reduce the cumulative emissions of N_2_O in the soil to a certain extent (Hao et al., 2023), because in the environment of conservation tillage, soil N_2_O emissions are inhibited, shortening the time of N_2_O emissions after fertilization and reducing the magnitude of emissions, and the strengthening of denitrification leads to the increase of N_2_O emissions in conventional tillage soil (Wang et al., 2021; Wang et al., 2022). This study found that, compared with other treatments, STS had a lower N_2_O emission flux after fertilization, and the cumulative emission was significantly lower than other treatments. This might be because subsoiling increases the aeration of the soil layer, which inhibits the respiration of anaerobic denitrifying bacteria, and it is difficult to form an anaerobic environment conducive to denitrification, resulting in a decrease in the intensity of denitrification in the soil, thereby reducing N_2_O emissions.

### 4.3 Effects of tillage methods on soil nitrogen supply capacity

Tillage practices affect soil N storage and crop availability. Studies have found that the N content of soil could be significantly reduced after tillage (Xiao et al., 2017, Xiao et al., 2019), which may be attributed to the increase of tillage intensity and frequency, which could accelerate the turnover of soil N and lead to the loss of soil N. In this study, the content of soil N fractions in 0–30 cm soil layer of STS was higher than that of PT, RT and ST treatments at jointing, anthesis and maturity (**Figure 8**). The disturbance of PT and RT on soil was large, which destroyed soil aggregates and accelerated the turnover of N. Compared with ST treatment, subsoiling not only had the characteristics of smaller soil disturbance, but also improved the soil aeration in deeper soil layers, which improved the soil microenvironment, promoted microbial activity, and was conducive to the increase of MBN content. In addition, subsoiling promoted root penetration, enhanced the absorption capacity of roots to deep soil nutrients, made roots grow vigorously, and then produced a large amount of secretions from roots, providing sufficient SON for plant absorption. NH_4_^+^-N and NO_3_^--^N, as available N sources for crop growth, are significantly affected by the N transformation process, especially in the 0–30 cm soil layer, which is positively correlated with soil ammonification and nitrification intensity (**Figure 11**). It may be that STS treatment has higher soil ammonification and nitrification intensity (**Figures 4B, C**), which promotes the conversion of organic N to NH_4_^+^-N and NO_3_^--^N in soil, and then provides sufficient N source for wheat growth and development. However, excess inorganic N that exceeds crop N demand increases the risk of environmental pollution caused by N loss (Chen et al., 2021). In this study, ^15^N isotope tracer technique was used to find that the soil N uptake was the largest in each tillage treatment. Compared with PT, RT and ST treatments, STS treatment obtained the highest N accumulation, with an increase of 4.0%-14.98% (**Figure 9**). In addition, STS treatment significantly increased the proportion of N fertilizer absorbed by wheat, and significantly reduced the proportion of N fertilizer loss, which was 10.71%-30.31% lower than other treatments (**Figure 10**). This further shows that STS treatment is beneficial to improve the absorption of N fertilizer by wheat, reduce the loss of N fertilizer, and promote the efficient use of N fertilizer. Through least squares multiplication, it was found that tillage methods significantly promote N transformation and N pool composition, indirectly promoting N absorption, thereby increasing wheat yield, while suppressing cumulative N_2_O emissions and reducing nitrogen loss. Especially, STS treatment increased nitrogen absorption and achieved the highest yield, indicating that STS treatment can achieve a synergistic improvement in nitrogen absorption and yield.

Although our analysis helps to improve our understanding of the process of soil N transformation and N supply capacity through long-term cultivation, some limitations should be noted. In this paper, the method of using different substrates for indoor culture to evaluate the potential of N transformation intensity may not accurately reflect the actual intensity or dominance of the specific N transformation process *in situ*. However, in the N cycle of agricultural ecosystems, microbial communities play a crucial role in driving N transformation through functional genes such as amoA, nirS/nirK, and nosZ. Therefore, it is crucial to analyze the microbial characteristics of soil N transformation in the future to clarify the mechanism of long-term tillage on soil N transformation at a deeper level, and to provide a more solid theoretical basis for tillage practices that reduce N loss and improve N use efficiency.

## 5 Conclusion

This study evaluated the long-term effects of four tillage methods on soil N transformation process and soil N fractions at different growth stages of winter wheat for 16 years. The results showed that soil N transformation intensity, soil N-transforming enzyme activity and soil N fractions changed with the growth of wheat. Compared with PT, RT and ST, STS treatment increased soil nitrogen fixation, ammonification, and nitrification intensity in 0–30 cm soil layer at different growth stages, enhanced the activities of urease and protease, which promoted the transformation of soil N, but significantly reduced soil denitrification intensity, the nitrate reductase and nitrite reductase activities, reduced the accumulation of N_2_O, and further reduced the loss of N. At the same time, STS treatment had higher soil N content, which provided sufficient N supply for wheat growth. In addition, STS treatment increased N absorption and reduced N loss, further improving grain yield and yield stability. The least squares method reveals that STS promotes soil N transformation and increases N pool composition, indirectly promoting N absorption, thereby increasing wheat yield, while suppressing cumulative N_2_O emissions and reducing nitrogen loss.In general, STS treatment promoted soil N transformation, reduced soil N loss, provided sufficient N supply for plant growth, promoted plant N absorption, and obtained the highest grain yield. It is a sustainable tillage strategy to reduce N loss and increase yield. In the future, further research on the role of microorganisms in soil N transformation is crucial for understanding the relationship between soil N transformation and N supply capacity. This result can provide a sustainable farming strategy for reducing N loss and increasing yield in the Huang-Huai-Hai region. However, there are still key gaps in understanding how key microorganisms and specific N cycle genes in the process of soil N transformation under long-term tillage conditions affect soil N components and their interactions with soil environmental factors. Future research should focus on exploring the critical role of microorganisms in soil nitrogen cycling under long-term cultivation conditions to support global sustainable agriculture and efficient nutrient management.

## Data Availability

The original contributions presented in the study are included in the article/supplementary material. Further inquiries can be directed to the corresponding author/s.
